# Breaking New Ground: The Crucial Role of Animal Research in the Advancement of Rhabdomyolysis-Induced AKI Treatment and Prevention

**DOI:** 10.1093/function/zqad039

**Published:** 2023-07-26

**Authors:** Marharyta Semenikhina, Joshua H Lipschutz, Oleg Palygin

**Affiliations:** Division of Nephrology, Department of Medicine, Medical University of South Carolina, Charleston, SC 29425, USA; Division of Nephrology, Department of Medicine, Medical University of South Carolina, Charleston, SC 29425, USA; Ralph H. Johnson Veterans Affairs Medical Center, Charleston, SC 29401, USA; Division of Nephrology, Department of Medicine, Medical University of South Carolina, Charleston, SC 29425, USA

## A Perspective on “Post-Injury Inhibition of Endothelin-1 Dependent Renal Vasoregulation Mitigates Rhabdomyolysis-Induced Acute Kidney Injury”

The breakdown of damaged muscle tissues leads to the release of various substances, including myoglobin, potassium, creatine kinase, and lactic acid, into the bloodstream. Unfortunately, this can result in acute kidney injury (AKI), which is a major risk factor for mortality, chronic kidney disease (CKD), and end-stage kidney disease (ESKD). This dangerous condition, known as rhabdomyolysis-induced acute kidney injury (RIAKI), is a common complication of acute skeletal muscle destruction, affecting anywhere from 13% to 50% of patients.^1^ Rhabdomyolysis can be caused by a range of factors, either on their own or in combination, such as trauma, prolonged immobilization, intense physical activity, certain medications, infections, and even hereditary or acquired metabolic myopathies. Historically, detailed reports of rhabdomyolysis symptoms and the understanding of its renal complications emerged from a large cohort of combat casualties during World Wars I and II, when the basic mechanisms of RIAKI and harmful impacts of myoglobinuria on kidney function were established.^2^ Since then, RIAKI has remained an important topic in civilian disasters and military medicine and continues to be a challenging issue contributing to morbidity and mortality.^3^

Myoglobin is a cytoplasmic protein of skeletal and cardiac muscle that binds oxygen in a heme group and facilitates tissue oxygen diffusion. The release of myoglobin into the bloodstream in the context of rhabdomyolysis is one of the main factors causing renal dysfunction. As a 17.8 kDa protein, myoglobin passes the glomerular filtration barrier and gets concentrated along the renal tubules. Renal myoglobin promoting oxidative and nitrosative stress in the proximal tubule also leads to granular casts and obstruction in the distal parts of nephron. The pathological shift in redox balance mediated by myoglobin results in nitric oxide deficiency and lipid peroxidation, which triggers renal vasoconstriction. Together with other factors, such as rhabdomyolysis-associated activation of the renin angiotensin aldosterone system (RAAS), volume depletion, and release of vascular mediators [for instance, endothelin-1 (ET-1) and TNF-α], this cascade of events promotes pathological remodeling on both endothelial and epithelial cells and leads to AKI.^4^

The diagnosis of RIAKI is based on the detection of muscle damage markers such as elevated creatine kinase (CK) levels, myoglobinuria, aspartate aminotransferase (AST), serum phosphate, and electrolyte abnormalities. The latter may include hyperkalemia, hypocalcemia, metabolic acidosis, and play a significant role in the development and severity of renal injury. The prevention and treatment protocol for RIAKI consists mainly of supportive therapy aimed at preserving kidney function, and may include volume repletion, electrolyte, pH and osmolarity corrections, and renal-replacement therapy. The development of new effective therapeutic strategies for RIAKI is sorely needed and would rely on an expanded understanding of the mechanisms underlying the regulation of renal electrolyte transport, vasoconstriction and vascular mediator-associated pathways, mitochondrial bioenergetics, and oxidative stress.

Animal models have been successfully used to address several key questions that improved our understanding of RIAKI mechanisms. Currently, animal studies of RIAKI are based on glycerol-induction, crush injury, ischemia/reperfusion, myoglobin injection, as well as alcohol or drug-induced models of rhabdomyolysis.^5,6^ The most common model uses an intramuscular or intravenous injection of glycerol to promote muscle damage and subsequent release of muscle cell contents into the bloodstream. The crush injury-induced rhabdomyolysis model applies external pressure or crushing force to a specific muscle (ie, quadriceps, gastrocnemius, soleus muscles, and extensor digitorum longus). Similarly, nonsurgical physically induced injury can be established by force applied to the muscle, which leads to the breakdown of muscle fibers and release of myoglobin. The severity of the injury in this model is adjusted based on the duration and intensity of the applied pressure and can vary depending on the protocol. The ischemia/reperfusion-induced rhabdomyolysis model is an experimental approach based on temporary interruption of blood flow by occluding the blood vessels supplying a specific muscle, followed by reperfusion. This model mimics vascular occlusion and temporary ischemia. With an injury, in the absence of oxygen and energy, muscle cells switch to anaerobic metabolism resulting in the production of lactic acid and other metabolites, which following reperfusion leads to ROS production and triggers an inflammatory response. The combination of ischemia and reperfusion results in extensive muscle cell damage and rhabdomyolysis. Other approaches employ a direct injection of myoglobin or muscle extracts containing myoglobin into the bloodstream, where myoglobin can then reach the kidneys and promote AKI.^5^ Alcohol-induced rhabdomyolysis includes prolonged (3–4 wk) exposure to alcohol and food deprivation in animals resulting in elevation of serum CK, myoglobinuria, and muscle fiber necrosis. Drug-induced models of rhabdomyolysis were recently introduced and are based on statins’ (also known as HMG-CoA reductase inhibitors, which are a common cholesterol-lowering medication) side effects of rhabdomyolysis.^7^

**Figure 1.**
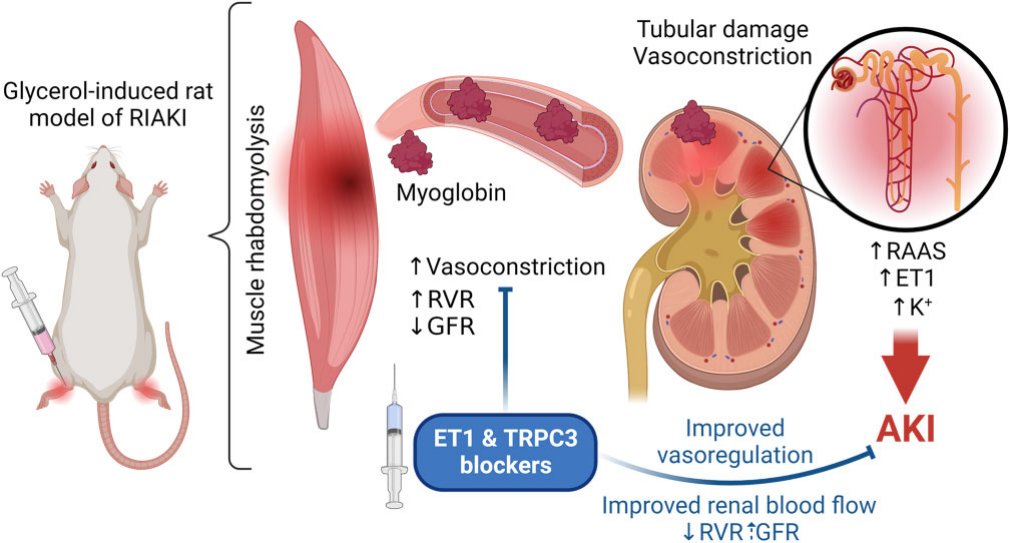
Endothelin-1 and TRPC3 contribute to renal vascular reactivity and RIAKI. Glycerol-induced muscle rhabdomyolysis promotes vasoconstriction, increases ET-1 production and RVR, as well as decreases GFR, which culminates in AKI. Endothelin-1 and TRPC3 significantly contribute to the dysregulation of renal hemodynamics in RIAKI. Moreover, attenuation of these factors in TRPC3 knockout rats and through pharmacological interventions effectively attenuates the pathological alterations in vasoregulation and renal function, offering potential therapeutic strategies for RIAKI management.

The abovementioned animal models played an important role in the development of RIAKI treatments. As an example, the protective effects of lipid peroxidation inhibitors^8^ and the osmotic diuretic mannitol^9^ during rhabdomyolysis were established using a glycerol-induced myohemoglobinuric AKI rat model. The recent study of Afolabi et. al.^10^ conducted on Wistar rats utilized this approach to explore the mechanisms behind rhabdomyolysis-mediated vasoconstriction and its effects in RIAKI. Endothelins are potent regulators of vascular tone and ET-1 has a significant impact on vascular function through the regulation of ion channels such as the transient receptor potential cation channel, subfamily C member 3 (TRPC3). Afolabi et al. demonstrated that glycerol-induced rhabdomyolysis leads to increased production of ET-1, which in turn elevates renal vascular resistance (RVR). This results in a decline in glomerular filtration rate (GFR) and exacerbates AKI. However, the study also presented a potential solution to this problem. By pharmacologically inhibiting the ET-1 pathway and the TRPC3 signaling cascade after injury, the researchers observed a significant attenuation of pathological changes, improvement in vasoregulation, and mitigation of AKI (as depicted in   Figure 1). The authors of the study suggested that the production of ET-1 and the activation of TRPC3-dependent renal vasoconstriction play a critical role in the development of rhabdomyolysis-induced AKI. Therefore, they proposed that targeting ET-1-mediated renal vasoregulation following injury could be a promising therapeutic approach for the treatment and prevention of renal damage caused by rhabdomyolysis.

In the ever-evolving landscape of medical research, the field of RIAKI has seen remarkable advancements. Yet, amidst this progress, numerous crucial questions remain unanswered. The latest findings on proximal tubule redox imbalance and ferroptotic cell death, as well as the regulation of potassium transport in distal tubules, the myopathic form of CPT II deficiency, and ion channel pharmacology present exciting opportunities to find better ways to prevent RIAKI-associated complications such as oxidative stress, hyperkalemia, and metabolic disorders. By integrating these findings with existing animal models, future studies have the potential to enhance our understanding of RIAKI pathology and, importantly, foster the development of groundbreaking clinical tools.
